# Capacity building in national influenza laboratories – use of laboratory assessments to drive progress

**DOI:** 10.1186/s12879-015-1232-1

**Published:** 2015-11-06

**Authors:** Lucinda E. A. Johnson, Sarah A. Muir-Paulik, Pam Kennedy, Steven Lindstrom, Amanda Balish, Tricia Aden, Ann C. Moen

**Affiliations:** Influenza Division, National Center for Immunization and Respiratory Diseases, Centers for Disease Control and Prevention, Atlanta, GA 30333 USA; Association of Public Health Laboratories, Silver Spring, MD USA; McKing Consulting Corporation, Fairfax, VA USA; Battelle Memorial Institute, Columbus, OH USA

**Keywords:** Influenza, Laboratories, Capacity building, Surveillance, IHR, Global health

## Abstract

**Background:**

Laboratory testing is a fundamental component of influenza surveillance for detecting novel strains with pandemic potential and informing biannual vaccine strain selection. The United States (U.S.) Centers for Disease Control and Prevention (CDC), under the auspices of its WHO Collaborating Center for Influenza, is one of the major public health agencies which provides support globally to build national capacity for influenza surveillance. Our main objective was to determine if laboratory assessments supported capacity building efforts for improved global influenza surveillance.

**Methods:**

In 2010, 35 national influenza laboratories were assessed in 34 countries, using a standardized tool. Post-assessment, each laboratory received a report with a list of recommendations for improvement. Uptake of recommendations were reviewed 3.2 mean years after the initial assessments and categorized as complete, in-progress, no action or no update. This was a retrospective study; follow-up took place through routine project management rather than at a set time-point post-assessment. WHO data on National Influenza Centre (NIC) designation, External Quality Assessment Project (EQAP) participation and FluNet reporting was used to measure laboratory capacity longitudinally and independently of the assessments. All data was further stratified by World Bank country income category.

**Results:**

At follow-up, 81 % of 614 recommendations were either complete (350) or in-progress (145) for 32 laboratories (91 % response rate). The number of countries reporting to FluNet and the number of specimens they reported annually increased between 2005, when they were first funded by CDC, and 2010, the assessment year (*p* < 0.01). Improvements were also seen in EQAP participation and NIC designation over time and more so for low and lower-middle income countries.

**Conclusions:**

Assessments using a standardized tool have been beneficial to improving laboratory-based influenza surveillance. Specific recommendations helped countries identify and prioritize areas for improvement. Data from assessments helped CDC focus its technical assistance by country and region. Low and lower-middle income countries made greater improvements in their laboratories compared with upper-middle income countries. Future research could include an analysis of annual funding and technical assistance by country. Our approach serves as an example for capacity building for other diseases.

## Background

Influenza is associated with considerable morbidity and mortality worldwide; an estimated 3–5 million cases of severe disease and 250,000-500,000 deaths annually [1]. During a pandemic this disease burden can increase significantly [2–4]. Prevention and control of influenza is dependent on adequate surveillance via the World Health Organization (WHO) Global Influenza Surveillance and Response System (GISRS) [1]. The United States (U.S.) Centers for Disease Control and Prevention (CDC) is a WHO Collaborating Centre for the Surveillance, Epidemiology and Control of Influenza. In this capacity, CDC provides support globally, to build sustainable national capacity for influenza surveillance, enhance participation in WHO GISRS and build response capacity for the WHO International Health Regulations (IHR, 2005) [5].

CDC influenza support is provided in the form of funding and technical assistance to national ministries of health through bilateral cooperative agreements. An important goal of this bilateral support is to help the national influenza laboratories of countries attain or maintain WHO National Influenza Center (NIC) status and standards of practice. A critical function of NICs within GISRS, the global alert system for influenza viruses with pandemic potential, is to test and ship unusual influenza specimens to WHO Collaborating Centers (WHO CCs) for further analysis and identification [6]. NICs also share circulating seasonal viruses with WHO CCs to inform vaccine strain selection. NICs are designated by ministries of health and recognized by WHO upon confirmation that they meet the NIC terms of reference [6].

In 2009, CDC and the Association of Public Health Laboratories (APHL) developed the *International Influenza Laboratory Capacity Review* tool [7] (hereafter referred to as the ‘Tool’) as a means to identify the capacity of national influenza laboratories [8]. The Tool is composed of a series of questions, which cover a range of general and influenza-specific laboratory functions such as, virology and biosafety, as well as the quality of those functions. It is completed during country site visits by influenza experts from U.S. public health laboratories (including CDC) who make recommendations for improvement based on their observations and responses to the questions. Site visits or ‘assessments’ also provide an opportunity to deliver technical assistance, and to identify the resource requirements for each laboratory. The overall goal of the Tool, and each assessment, is to improve the quality of the work being done within GISRS and in particular, to accelerate national influenza laboratories towards achieving NIC recognition where it does not yet exist.

Here we report the outcomes from 35 national influenza laboratory assessments from 34 countries which took place between September 2009 and December 2010. We describe the use of a standardized tool which was developed to guide the assessment process and lend support to improvements in laboratory-based influenza surveillance. Data from WHO is presented, including participation in GISRS, as a means to independently measure the influenza testing capacity of laboratories. Finally, to understand the technical assistance and funding needs of countries, the results were examined by World Bank country income category.

## Methods

For a detailed description of the development of the *International Influenza Laboratory Capacity Review* tool [7] including the assessment process, the addition of a quantitative component and the total number of assessments conducted to August 2014, please refer to Muir-Paulik et. al. [8]

### Ethics statement

This study did not constitute human subjects research; data about a human that cannot be linked back to the human is exempt [9]. Our data sources included 1) information collected during assessments of laboratories which was in a manner that subjects cannot be identified and 2) information from existing, publicly available data sets. Assessments are voluntary and are offered to countries supported by CDC or at the request of WHO. Laboratories are informed that all data collected through assessments is confidential and will only be used in aggregate unless permission is specifically requested to use individual country results.

### Laboratory assessment countries

Countries participating in assessments included Angola, Argentina, Armenia, Brazil, Cambodia, Congo, Côte d’Ivoire, the Democratic Republic of Congo, Egypt, Ethiopia, Fiji, Georgia, Indonesia, Kenya, Laos, Mexico, Moldova, Mongolia, Morocco, Nepal, Nicaragua, Nigeria, Panama, Paraguay, Peru, Philippines, Rwanda, Sri Lanka, Tanzania, Thailand, Uganda, Ukraine, Vietnam and Zambia. Vietnam has two national influenza laboratories both of which were assessed and included in this analysis.

### Laboratory capacity tool

The *International Influenza Laboratory Capacity Review* tool [7] is a document containing a series of questions covering both general and influenza-specific functions, including the quality of those functions. These questions aim to identify a laboratory’s accuracy and reliability of test results, timeliness and frequency of testing and reporting, representativeness of sampling, presence and maintenance of equipment, professional development of staff, implementation of biosecurity measures and safe handling and containment of infectious microorganisms and hazardous biological materials.

### Assessors

Assessors are laboratory experts from U.S. public health laboratories (including CDC). At the time of the first assessments there was a pool of approximately 15 assessors. Some assessors went to several laboratories while others visited just one or two. All assessors were provided with group training prior to conducting their first assessment to help achieve standardization across the assessments. In addition, each new recruit was accompanied by an experienced assessor during their first on-site laboratory assessment. Lastly, inter-rater reliability exercises have since been added to trainings and a user guide with definitions for each question was developed to enhance standardization across reviews.

### Assessment process

One assessment took place in September 2009 but 34 took place in 2010, so we refer to the assessment year as 2010. Each assessment took place over a three to five day period starting with a briefing to describe the purpose and process of the assessment for host-country government representatives and influenza laboratory staff. The assessors then used the Tool to document their findings and guide their discussions with laboratory staff as they made direct observations of laboratory operations. As needed, assessors provided hands-on technical assistance during assessments and addressed any questions. Upon completion, assessors de-briefed laboratory staff and key stakeholders with a description of their findings and any major recommendations. A detailed written report listing the laboratory’s strengths and recommendations for improvement was shared with each laboratory for input before being finalized. Reports are confidential and shared only with the country being assessed.

Through routine project management rather than at a designated time point, but at least one year after assessments were conducted, the uptake of recommendations by laboratories was assessed. For some laboratories this was accomplished during a repeat, on-site assessment of the laboratory as described above, for other laboratories it was completed through correspondence by telephone and/or email between CDC project officers and laboratory staff.

### Recommendations analyses

Recommendations made for the 35 assessments were reviewed and categorized as general, procurement, or training. Examples of general recommendations included, ensuring real-time polymerase chain reaction (PCR) machines are calibrated annually, and recording surveillance data and test results electronically. Examples of procurement recommendations included, purchasing certified thermometers, and ordering properly fitting pipette tips for micropipettes. Examples of training recommendations included, teaching staff real-time PCR for influenza virus detection and subtyping, and certifying staff to package infectious materials for international shipping.

At follow-up, recommendations were categorized as: complete, in-progress, no action or no update. The data were aggregated for all the laboratories by recommendation type (general, procurement or training) and status update. Results were also stratified by World Bank income category [10] and analyzed by outcome using the binomial exact test because our sample sizes were small.

### WHO FluNet reporting, EQAP participation and NIC designation

Data was extracted from the WHO influenza virological surveillance database, FluNet [11], to determine which of the 34 countries were reporting influenza data between January 1, 2005, the year in which countries first received CDC support, and December 31, 2010, the year in which laboratory assessments took place. The binomial exact test was used to compare the number of participating countries reporting by World Bank income category [10]. The Student *T*-test was used to compare the number of influenza specimens reported by the countries over the same period. Since the FluNet database is publicly available, we have presented data from the 34 countries through to the end of 2013 to show the current level of participation.

We were provided with data from WHO regarding participation of the 35 laboratories in the WHO External Quality Assessment Project (EQAP) for the detection of influenza virus type A by PCR [11] in 2007 (when the EQAP began), and 2010. Under this program, laboratories receive a panel of circulating influenza specimens to identify, as well as a questionnaire on good laboratory practices. Data from WHO was also used to examine the WHO NIC status of each laboratory in 2005 and 2010. The binomial exact test was used to compare the outcomes between 2007 and 2010 for EQAP participation and between 2005 and 2010 for NIC status.

## Results

### Laboratory assessments & recommendations analyses

We conducted and reviewed 35 assessments in 34 countries in 2010. We were able to follow up recommendations made to 32 laboratories in 31 countries, a 91 % response rate; two of the countries stopped participating in their cooperative agreements and one country did not provide follow-up data. A total of 614 recommendations were made to these 32 laboratories in 2010; 334 (54 %) general, 185 (30 %) procurement and 95 (15 %) training (Table 1). Follow up to determine the status of recommendations took place a mean of 3.2 ± 0.8 years after laboratory assessments were conducted. The majority of recommendations (81 %) were either complete (350) or in-progress (145) at follow-up. It was found that 35 (10 %) general recommendations were not implemented (categorized as “no-action”) compared with 49 (26 %) and 21 (22 %) for procurement and training recommendations, respectively.

**Table 1:** Recommendations from 32 national laboratory assessments in 31 CDC partner countries and their follow-up status

Recommendations (2010)		Follow-up Status (average of 3.2 years from 2010)			
Category	Number (%)	Complete	In-progress	No Action	No Update
General	334 (54 %)	205 (61 %)	87 (26 %)	35 (10 %)	7 (2 %)
Procurement	185 (30 %)	99 (54 %)	32 (17 %)	49 (26 %)	5 (3 %)
Training	95 (15 %)	46 (48 %)	26 (27 %)	21 (22 %)	2 (2 %)
All	614	350 (57 %)	145 (24 %)	105 (17 %)	14 (2 %)

Of the 34 countries assessed, eight (23.5 %) are classified by the World Bank as low-income economies, 18 (53 %) as lower-middle income economies and eight (23.5 %) as upper-middle income economies. When the recommendations data was analyzed by World Bank income category the results were only significant for one result; laboratories from lower-middle income countries completed more training recommendations when compared with laboratories from upper-middle income countries (*p* < 0.01).

### WHO FluNet reporting, EQAP participation and NIC designation

Statistically significant improvements in WHO FluNet reporting, EQAP participation and NIC designation were seen among countries/laboratories between 2005 and 2010 (Table 2). The increase in FluNet reporting among low and lower-middle income countries between 2005 and 2010 was statistically significant (*p* < 0.01) (Table 2). Likewise, laboratories in low and lower-middle income countries saw a statistically significant increase in EQAP participation between 2007 and 2010 (*p* < 0.01) (Table 2). NIC designation increased significantly for laboratories from lower-middle income countries between 2005 and 2010 (*p* < 0.05) (Table 2).

**Table 2:** WHO FluNet reporting, NIC designation and EQAP participation for 35 national laboratories, 2005/2007 and 2010

Country/Laboratory World Bank Income Category	# Countries reporting to FluNet		# Laboratories with an NIC		# Laboratories participating in EQAP	
	2005	2010	2005	2010	2007	2010
Low (*n* = 8)	0 (0 %)	7 (88 %)**	2 (25 %)	4 (50 %)	2 (25 %)	8 (100 %)**
Lower-middle (*n* = 18/19)	7 (39 %)	17 (94 %)**	7 (37 %)	14 (74 %)*	11 (58 %)	18 (95 %)**
Upper-middle (*n* = 8)	5 (63 %)	8 (100 %)	6 (75 %)	7 (88 %)	5 (63 %)	7 (88 %)
Total	12 (35 %)	32 (94 %)**	15 (43 %)	25 (71 %)*	18 (51 %)	33 (94 %)**

*Difference between 2005 and 2010 is statistically significant at *p* < 0.05 using the binomial exact test

**Difference between 2005/2007 and 2010 is statistically significant at *p* < 0.01 using the binomial exact test

Changes in WHO FluNet reporting, National Influenza Centre designation and External Quality Assessment Project participation for 35 national laboratories in 34 CDC partner countries between 2005 and 2010

**Fig. 1:**
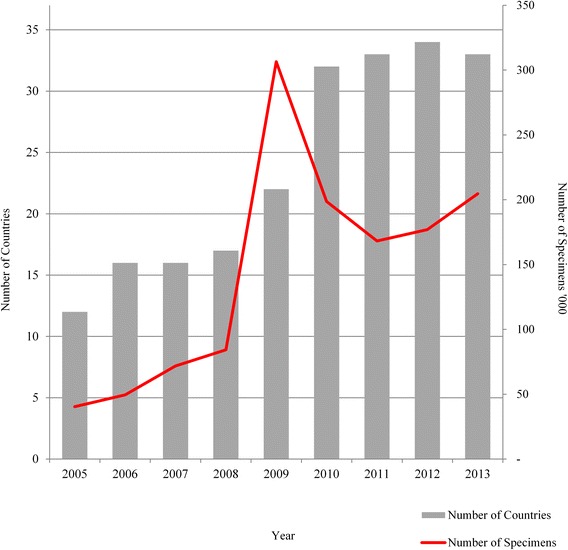
WHO FluNet reporting between 2005 and 2013. Number of countries reporting to WHO FluNet between 2005 and 2013 and number of specimens reported annually

The total number of specimens reported to FluNet from participating countries increased from 40,406 in 2005 to 198,704 in 2010 (*p* < 0.01) (Fig. 1). By the end of 2013 there were additional improvements; 33 of the 34 countries (97 %) reported a total of 204,701 specimens to FluNet. The peak in 2009 of 306,430 specimens represents increased activity associated with the influenza A (H1N1) 2009 pandemic.

## Discussion

Assessments using a standardized tool have been beneficial to improving laboratory-based influenza surveillance. Providing a list of recommendations for improvement has helped countries to identify, prioritize and advocate for training, funds, supplies and other resources to meet the needs of their national influenza laboratories. For example, in one laboratory, the floors and ceilings were cracked and moldy due to water leakages, causing a safety hazard. This country used its assessment report to obtain government funding to make the recommended repairs. The high uptake of recommendations observed (either complete or in-progress) is suggestive of improved laboratory practices and, by implication, enhanced surveillance.

A greater proportion of general recommendations were complete or in-progress at follow-up when compared with procurement and training recommendations. This may be the result of funding constraints, since all participating laboratories were located in low- and middle-income countries. Most procurement recommendations required expenditure, for example, purchasing equipment. Likewise, staff training is likely to require financial resources for travel, to send staff overseas, or bring experts into a country. By comparison, many general recommendations required a change in laboratory practices or procedures needing little or no funding. For example, altering an influenza testing algorithm or updating written standard operating procedures (SOPs) requires staff time and expertise, but not expenditure and may therefore have been easier to implement.

The assessments have helped determine how to best work with participating laboratories to improve influenza testing and surveillance. For example, an analysis of the first 26 assessments helped determine that training in laboratory management, especially quality assurance (QA) and biosafety, were needed most among African countries. As a consequence, CDC, APHL and the National Institute for Communicable Diseases in South Africa developed and delivered the course “Improving Influenza Laboratory Management Practices” in Johannesburg in early 2011. This course covered a broad range of topics, and was subsequently tailored to regional training conducted in Bangkok in March 2012 and Istanbul in May 2014. These three courses have provided training to a total of 112 participants from 59 countries.

The assessments have served to build laboratory capacity by offering an opportunity for the provision of real-time technical assistance. As the assessors worked through the Tool and made their observations, they were able to identify immediate areas of concern, address questions from staff, and troubleshoot procedures. For example, assessors and staff discussed ways that laboratory equipment could be rearranged or, space reassigned to create a unidirectional workflow for PCR to minimize contamination. Types of troubleshooting included addressing poor PCR efficiency and inadequate growth of cells for tissue culture and virus isolation. During one site visit, an assessor set up a new piece of equipment for the automated extraction and purification of influenza RNA, which was awaiting installation. Staff were trained in its use, saving valuable time in sample preparation, which could be critical in the case of an epidemic.

While laboratory testing is considered essential to disease diagnostics and surveillance, it is also recognized as a weak link in public health systems in resource-challenged settings [12–16]. Studies which have examined laboratory capacity building in such settings, point to a number of barriers to improvement, including but not limited to, poor infrastructure, lack of qualified laboratory staff, insufficient maintenance of equipment, poor internal QA and limited written SOPs. [12, 14, 16–21]. Many of these challenges were also present in the laboratories assessed here, which was evident in the types of recommendations made. For example, 20 laboratories received at least one recommendation regarding the development of written SOPs while 30 laboratories received at least one for QA. Among the QA suggestions, 11 laboratories were recommended to regularly monitor the temperature of freezers and refrigerators where critical reagents were stored. Other QA recommendations included separating pre- and post-PCR work, calibrating equipment, aliquoting reagents, and using controls in experiments.

Quality systems are critical to ensuring strong laboratory capacity; however, demonstrating accuracy and reliability of testing is a major challenge in resource-limited countries [12, 15, 18]. Within the context of laboratory-based influenza surveillance, designation as a WHO NIC is considered an important goal for demonstrating quality systems; NIC status indicates the capacity of a laboratory to meet the WHO’s minimum terms of reference to identify, analyze and share virus isolates. The number of laboratories designated as NICs substantially increased between 2005 and 2010, which reflects an improvement in quality systems among participating countries despite being low and middle-income economies. This finding also supports the assumption that CDC funding and technical assistance received by the 34 countries over this period helped to improve influenza laboratory capacity.

Participation in external quality assessments is lacking in many laboratories in resource-challenged settings [12, 15, 18]. By comparison, in 2010, most of the national influenza laboratories which were assessed, participated in the WHO EQAP for the detection of influenza virus type A by PCR [11]. Participation increased substantially between 2007 and 2010, indicative of improved diagnostic capacity among the laboratories since testing requires PCR equipment and expertise. EQAP participation was an explicit recommendation to countries prior to 2010 and during the assessments. Although it does not reveal the proficiency of testing, the increase in EQAP participation observed helps to verify recommendations were implemented and indicates that laboratories are committed to making improvements in the quality of their testing.

The number of countries submitting data to the WHO virological database, FluNet, as well as the number of specimens submitted increased from 2005, the year in which countries were first funded by CDC, to 2010, when the assessments took place. While the increase may be partially attributed to enhanced surveillance during the influenza A (H1N1) 2009 pandemic, there is a clear upward trend prior to 2009, which continued after this event to 2013 (Fig. 1). These increases are a positive indicator of influenza testing capacity and evidence of engagement in GISRS among CDC partner countries. They also suggest that CDC support has assisted laboratory improvements. Reporting to FluNet, including the number of specimens processed, has continued to improve through to 2013, which independently supports the fact that recommendations made to laboratories have led to enhanced capacity building.

Until recently, laboratory capacity building has received relatively little funding when compared with the financial resources donor agencies have allocated to disease prevention, control and treatment [22, 23]. Furthermore, funding has largely been targeted to testing for HIV, malaria and tuberculosis [24–26]. In the analysis of longitudinal data for EQAP participation, NIC designation and FluNet reporting, greater improvements were found among low and/or lower-middle income countries compared with upper-middle income countries given the same financial support and technical assistance provided by CDC to all partner countries. From 2005, countries received an annual median of 325,000 USD for influenza surveillance (both epidemiological- and laboratory-based) for a median of five years. These findings suggest that despite in-country financial constraints and modest CDC funding, targeted technical assistance, such as laboratory assessments with specific recommendations, can lead to substantial capacity building improvements.

There are some limitations to our study. We have inferred that funding and technical assistance provided by CDC between 2005 and 2010 has contributed to the improvement in capacity outcomes observed, but additional data and further analysis would be required to demonstrate this with greater confidence. Furthermore, we acknowledge the larger WHO GISRS and WHOCCs, in addition to other organizations, which have also contributed substantially to capacity building efforts. Our analysis of results by country income level may be limited to some extent by the small number of countries in the low income and upper-middle income categories; it is harder to show statistically significant improvements in these categories, especially among the upper-middle income countries which were performing better at baseline than low- and lower-middle income countries.

The importance of global capacity for influenza testing was demonstrated during the influenza A (H1N1) 2009 pandemic. In the early stages of this pandemic, GISRS facilitated the rapid sharing and analysis of virological specimens, allowing the WHO CC at CDC to quickly develop a diagnostic test which was then shared via the GISRS and Influenza Reagent Resource (IRR) websites [27]. Data from GISRS on circulating strains was used to establish vaccine candidates from which the pandemic vaccine was developed [27]. GISRS also played a pivotal role in mapping the spread of the virus globally [27]. GISRS, as well as the WHO’s influenza EQAP, has served as a model for improving laboratory surveillance for other IHR notifiable diseases, for example, Middle East respiratory syndrome coronavirus [28] and dengue fever [29].

## Conclusions

National laboratories are critical to ongoing global influenza surveillance. The U.S. CDC, as a WHO CC for influenza, has aimed to enhance this surveillance by improving the participation of its partner countries and their national laboratories in WHO GISRS. Laboratory assessments, conducted using a standardized tool and completed with specific recommendations, have been beneficial for building influenza laboratory capacity to this end. As a consequence, countries have also been able to make progress towards meeting their obligations under the IHR (2005).
